# Dendritic cell subsets in the intestinal lamina propria: Ontogeny and function

**DOI:** 10.1002/eji.201343740

**Published:** 2013-08-21

**Authors:** Emma K Persson, Charlotte L Scott, Allan McI Mowat, William W Agace

**Affiliations:** 1 Immunology Section Department of Experimental Medical Science, Lund UniversityLund, Sweden; 2 Centre for Immunobiology Institute of Infection Immunity and Inflammation, University of GlasgowGlasgow, Scotland, UK; 3 Department of International Health Immunology and Microbiology, Copenhagen UniversityCopenhagen, Denmark

**Keywords:** Antigen tolerance, Dendritic cells, Intestine, Lamina propria, Mucosa

## Abstract

The intestinal mucosa is exposed to large amounts of foreign antigen (Ag) derived from commensal bacteria, dietary Ags, and intestinal pathogens. Dendritic cells (DCs) are believed to be involved in the induction of tolerance to harmless Ags and in mounting protective immune responses to pathogens and, as such, to play key roles in regulating intestinal immune homeostasis. The characterization of classical DCs (cDCs) in the intestinal lamina propria has been under intense investigation in recent years but the use of markers (including CD11c, CD11b, MHC class II), which are also expressed by intestinal MΦs, has led to some controversy regarding their definition. Here we review recent studies that help to distinguish cDCs subsets from monocyte-derived cells in the intestinal mucosa. We address the phenotype and ontogeny of these cDC subsets and highlight recent findings indicating that these subsets play distinct roles in the regulation of mucosal immune responses in vivo.

## Introduction

Dendritic cells (DCs) are present throughout most tissues of the body where their main function is to sample self and foreign antigens (Ags), migrate to draining lymph nodes (LNs), and present antigenic peptides to naïve T cells, inducing their proliferation and differentiation. In so doing, DCs provide a critical link between the innate and adaptive immune systems. The intestinal lumen contains huge quantities of foreign Ag derived from commensal bacteria, dietary components, and occasional pathogenic microbes. Intestinal DCs are believed to play a central role in intestinal immune homeostasis, by inducing tolerance to harmless Ags, initiating protective immunity against intestinal pathogens, and contributing to intestinal diseases including celiac disease and the inflammatory bowel diseases (IBD, Crohn's disease, and ulcerative colitis).

Classical DCs (cDCs) are found throughout the intestinal lamina propria (LP), in gut-associated lymphoid tissue (GALT) including Peyer's patches (PPs) and isolated lymphoid follicles (ILFs), and in intestinal draining LNs such as the mesenteric LNs (MLNs). In the MLNs, cDCs can be broadly classified into lymphoid tissue resident cDCs, which are thought to derive from circulating precursors that enter directly from the blood, and migratory cDCs that arrive in organized lymphoid tissues via afferent lymph after acquiring Ag in the intestinal LP. While lymphoid tissue cDCs have been studied extensively, the functions of the intestinal cDCs that migrate to MLNs are less well understood.

The characterization of cDCs in the intestinal LP has been the subject of intense investigation and controversy, in part because the markers initially used to define them (including CD11c, CD11b and MHC class II (MHCII)) are also expressed by MΦs. In this review we discuss recent findings that have started to unravel the complexity of migratory intestinal cDCs, including assessment of the precursor-product relationships of transferred monocytes and cDC precursors, the identification of novel markers that appear to distinguish cDCs and monocyte-derived MΦs and new models that shed light on their in vivo functionality.

## Defining cDC subsets in the intestinal mucosa

**Figure 1:**
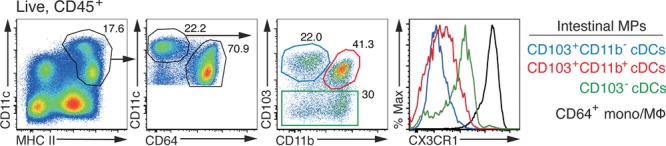
Phenotypic characterization of MPs in the SI LP. Representative flow cytometry plots of MPs in the steady-state SI LP of CX3CR1^gfp/wt^ mice (PPs are removed prior to analysis). Leukocytes are identified among live cells as CD45^+^. Monocytes/ MΦs are identified among the CD11c^+^MHCII^+^ cells as CD64^+^ cells, and cDCs are identified as CD64^−^ cells. cDCs can be separated into two major subsets of CD103^+^ cells: CD103^+^CD11b^+^ cDCs and CD103^+^CD11b^−^ cDCs, both of which are CX3CR1^lo/−^. In addition there is a minor population of CD103^−^ cDCs that express variable levels of CD11b, the majority of which are CX3CR1^int^. The CD11b^−^ cells within this latter population are not present in intestinal preparations of RORγt^−/−^ mice, which lack PPs and ILFs, suggesting they derive from GALT and not intestinal LP 5.

Although cDCs and other mononuclear phagocytes (MPs) such as monocytes and monocyte-derived MΦs in steady-state mouse LP can all express MHCII and CD11c, cDCs can be distinguished by their lack of expression of the high-affinity IgG receptor FcγR1, CD64 1,2 (Fig. 1). CD64^+^ MPs do not express the integrin α_E_ chain CD103 and include all CX3CR1^high^ cells that we, and others, have shown to be tissue resident MΦs 1–3. The CD64^+^ population also contains a subset of CX3CR1^int^ cells that co-express F4/80 and can be generated from adoptively transferred Ly6C^high^ monocytes. These cells represent intermediaries in the local differentiation of resident MΦs 1,2. In contrast, CD11c^+^MHCII^+^CD64^−^ MPs are F4/80^−^ and the majority of these cells express CD103. These CD103^+^ DCs have been held to be the prototypic migratory cDCs in the LP and can be divided into two main populations, CD103^+^CD11b^+^ and CD103^+^CD11b^−^ cDCs (Fig. 1). These subsets differ in their localization, transcription factor requirements, and function. Finally, there is a distinct population of CD11c^+^MHCII^+^CD103^−^ MPs in the intestine, which lack expression of the monocyte/MΦ marker CD64 4, and express heterogeneous levels of CD11b (Fig. 1). As will be discussed below, several lines of evidence suggest that these cells are bona fide migratory cDCs. Notably, intestinal CD103^−^CD11b^−^ but not CD103^−^CD11b^+^ cDC numbers are significantly reduced in RORγt^−/−^ mice that lack PPs and ILFs, suggesting that the former are GALT derived while the latter are resident within the intestinal LP 5.

## Ontogeny of intestinal cDC subsets

### Intestinal cDC precursors

According to current models, cDCs derive from committed cDC progenitors that develop in the bone marrow (BM) and travel via the blood to lymphoid organs and nonlymphoid tissues, where they differentiate into mature cDCs 6–9. Adoptive transfer studies have demonstrated that pre-cDCs, but not monocytes, give rise to both CD103^+^CD11b^+^ and CD103^+^CD11b^−^ cDCs in the intestinal LP 10,11. In addition, a subset of lineage^−^CD11c^+^α4β7^+^B220^+^CCR9^−^ cells termed premucosal DC progenitors was recently identified, which appeared, upon adoptive transfer, to home preferentially to the intestinal mucosa and give rise to both CD103^+^ cDC subsets 12. While these findings open up the intriguing possibility that there may be dedicated subsets of ‘gut-homing’ cDC progenitors, it is of note that these cells also gave rise to splenic cDCs (preferentially CD8α^+^ cDCs) and, in contrast to pre-cDCs, they also maintained the potential to develop into plasmacytoid DCs 12. Despite these findings, the relative contribution of different progenitor subsets to the development of the intestinal LP CD103^+^ cDC compartment in the steady state remains unclear. It is also unknown whether there are distinct precursors for CD103^+^CD11b^+^ and CD103^+^CD11b^−^ cDCs within these putative progenitor populations, or whether the final differentiation state of the precursors is determined by signals they receive within the intestinal environment. In this regard it is of interest to note that the relative proportion of CD103^+^CD11b^+^ and CD103^+^CD11b^−^ cDCs changes throughout the intestine, with CD103^+^CD11b^+^ cDCs comprising ∼70% of total CD103^+^ cDCs in the duodenum and CD103^+^CD11b^−^ cDCs comprising ∼75% of total CD103^+^ cDCs in the colon 13. CD103^+^CD11b^−^ cDCs dominate over CD103^+^CD11b^+^ cDCs in GALT 11, however, CD103^+^CD11b^−^ cDCs are present in normal numbers in the intestine of RORγt^−/−^ mice suggesting that they do not require GALT for their final differentiation and development 5.

The identity of the precursors of CD64^−^CD103^−^CD11b^+^ MPs remains unclear, as most studies that have assessed precursor differentiation in the intestine to date have not used CD64 or other appropriate markers to distinguish DCs from CD11b^+^ MΦs. As a result, it was frequently concluded that many or all CD103^−^CD11b^+^ MPs were derived from monocytes 3,10,11. However, two recent reports showed that adoptive transfer of Ly6C^high^ monocytes gave rise exclusively to F4/80^+^CD64^+^ MΦs and not CD64^−^CD11b^+^ MPs in the steady state, strongly indicating that the latter cells do not derive from monocytes 1,2. These findings may explain why a small population of CX3CR1^int^ cells was observed in the LP following transfer of pre-cDCs 10, although whether these cells lacked CD64 or F4/80 expression was not assessed in this study. More recently, systemic administration of anti-CCR2 antibody was shown to deplete CX3CR1^int^Ly6C^low^ cells expressing the DC-specific transcription factor Zbtb46 in DSS colitis 14. These findings are intriguing in view of the usual association of CCR2 with monocyte-derived MPs, and indicate the need for further studies of the progenitors of this subset of cDCs in the intestinal LP.

### Transcriptional factor requirements for intestinal cDC subsets

**Figure 2:**
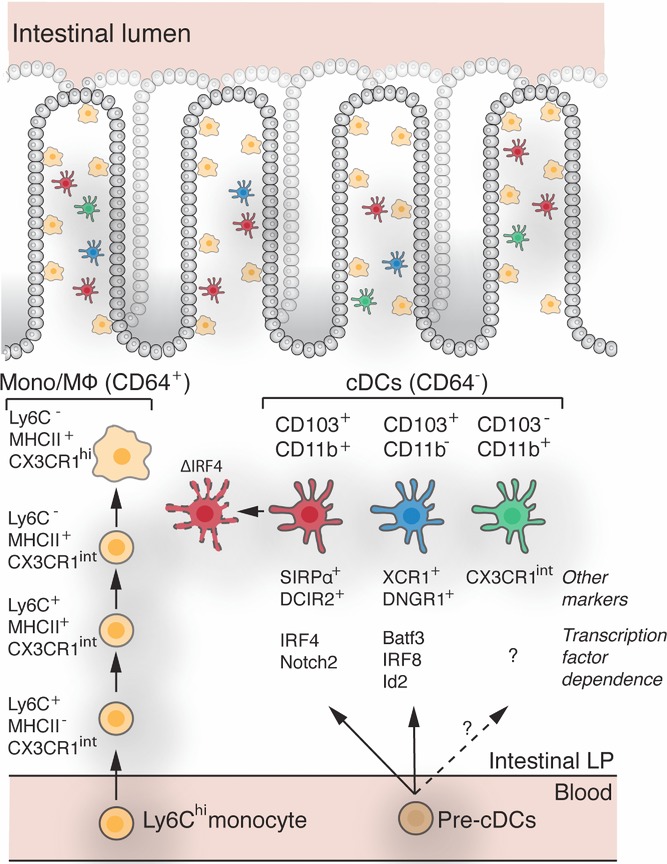
Overview of intestinal MPs. Ly6C^hi^ monocytes and cDC precursors seed the intestine from the blood where they give rise to distinct populations of MPs. cDC precursors, but not monocytes, give rise to CD103^+^CD11b^−^ and CD103^+^CD11b^+^ cDCs whose development/maintenance depends on distinct transcription factors. CD103^+^CD11b^−^ cDC are dependent on BatF3, IRF8, and Id2; CD103^+^CD11b^+^ cDC on IRF4 and Notch2. In the absence of IRF4 (ΔIRF4) CD103^+^CD11b^+^ cDC survival is compromised. It is currently unclear whether cDC precursors entering the intestine are precommitted to developing into one or other of these populations or whether the same precursor can develop into either subset depending on the signals it receives within the intestinal environment. In addition to CD103^+^ cDCs there is a minor population of CD103^−^CD64^−^CD11b^+^ cells in intestinal LP 4,5, which likely represent bona fide cDCs (see main text). In contrast to CD64^−^ cDCs, CD64^+^ MΦs develop from Ly6C^hi^ monocytes through a series of intermediate steps during which they lose the expression of Ly6C and gain expression of MHC class II 1,2.

It is now clear that the development of the intestinal CD103^+^CD11b^−^ and CD103^+^CD11b^+^ cDC subsets is dependent on distinct transcription factors. Peripheral tissue CD103^+^CD11b^−^ cDCs, including those located in the intestinal LP and PPs, are dependent on basic leucine zipper transcription factor ATF-like 3 (Batf3), inhibitor of DNA-binding 2 (Id2), and IFN-regulatory factor 8 (IRF8) 9,11,15–17 (Fig. 2). CD103^+^CD11b^−^ cDCs therefore appear developmentally linked to IRF8/Batf3-dependent lymphoid tissue resident CD8α^+^ cDCs 15,18,19. Supporting this idea, CD103^+^CD11b^−^ cDCs in LP express CD8α 5,20 and other markers typical of CD8α^+^ lymphoid cDCs, including the chemokine receptor XCR1 21,22 and the C-type lectin DNGR-1 23, and lack expression of DC inhibitory receptor 2 (DCIR2) 24 and signal regulatory protein (SIRP) α 5,24. Conversely CD103^+^CD11b^+^ intestinal cDCs develop independently of IRF8, Id2, and Batf3 9,16, and recent data from us, and others, suggest they may be developmentally related to splenic CD11b^+^CD4^+^ DCs 24,25. Both these populations of DCs express high levels of SIRPα and DCIR2 5,24, and lack expression of XCR1 21,22. Furthermore, we and others recently demonstrated that CD11c-Cre.IRF4*^fl/fl^* mice, whose DCs lack the transcription factor IRF4, referred to here as ΔIRF4 DC mice, have significantly reduced numbers of intestinal CD103^+^CD11b^+^ cDCs 4,24, as well as reduced numbers of splenic and LN resident CD11b^+^ DCs 24, together with a dramatic reduction in CD103^+^CD11b^+^ cDC numbers in intestinal draining MLNs 4,24. Intestinal CD103^+^CD11b^+^ cDCs isolated from ΔIRF4 DC mice died more rapidly in vitro compared with WT CD103^+^CD11b^+^ cDCs, and the few CD103^+^CD11b^+^ cDCs that remained in the MLNs of ΔIRF4 DC mice expressed Annexin V. Together these results indicate a key role for IRF4 in intestinal CD103^+^CD11b^+^ cDC survival (24, Fig. 2). Interestingly, mice with a DC-specific deletion in Notch-2 also display reduced numbers of both CD103^+^CD11b^+^ intestinal cDCs and a subset of splenic CD4^+^CD11b^+^ cDCs 25, providing additional evidence for a developmental link between these populations. CD11b^+^ lymphoid resident cDC homeostasis is also perturbed in TNF receptor-associated factor-6 (TRAF-6), IRF2, and Rel-B-deficient mice 26–28, but whether these factors contribute to intestinal CD103^+^CD11b^+^ cDC homeostasis remains to be explored. The transcription factor requirements for CD103^−^ cDCs remain to be determined.

### Growth factor requirements

cDC development and homeostasis are regulated by the growth factors FMS-like tyrosine kinase 3 ligand (Flt3L) and granulocyte MΦ colony-stimulating factor (GM-CSF/CSF-2) 29. The receptor for Flt3L (Flt3/CD135) is expressed on cDCs throughout their development, as well as on mature cells 7 and Flt3L induces cDC development from BM progenitors in vitro 30. Mice lacking the Flt3 receptor (Flt3^−/−^ mice) have a severe reduction in both CD103^+^CD11b^−^ and CD103^+^CD11b^+^ intestinal LP cDC numbers 11. While administration of exogenous Flt3L expands both subsets of intestinal CD103^+^ cDCs, it has a greater impact on intestinal CD103^+^CD11b^−^ cDCs 5,24 and DC-specific deletion of Pten, a negative regulator of Flt3L-induced PI3K-mTOR signaling, leads to increased numbers of intestinal CD103^+^CD11b^−^ cDCs 31. Collectively, these results demonstrate that Flt3L plays an important role in CD103^+^ cDC homeostasis. The role of CSF-2 in intestinal cDC development is less clear. Although utilized for generating human and murine DCs from blood monocytes and BM, respectively, in vitro 32, mice lacking CSF-2 or its receptor have normal numbers and proportions of LN DCs 33. Nevertheless, studies in CSF2R KO mice (Csf2rb^−/−^ and Csf2rb^−/−^ × Csf2rb2^−/−^), CSF-2 KO mice (CSF2^−/−^) and in WT mice following administration of exogenous CSF-2 have suggested that the homeostasis of intestinal CD103^+^CD11b^+^ DCs depends upon CSF-2 10,11,34. However, as CSF-2 can induce the expression of CD103 on cDCs 35,36 and cDCs from the lung and kidney of Csf2rb^−/−^ × Csf2rb2^−/−^ mice show a defect in CD103 upregulation 34, care needs to be exercised when using CD103 as a marker to define DCs in mice lacking CSF-2 function. To resolve this issue, future work on the role of CSF-2 in the development and homeostasis of intestinal DC subsets should use additional markers, such as XCR1, CD24, and CD8α, combined with CD11b or SIRPα, to define these subsets.

Whether CSF-2 or Flt3L is required for the development of the intestinal CD103^−^ cDCs is currently unclear. Administration of exogenous Flt3L to mice results in a significant expansion of both CD103^−^CD11b^−^ and CD103^−^CD11b^+^ cDCs in vivo 5 and it was recently reported that CD64^−^ DCs are virtually absent from the small intestine (SI) of Flt3L^−/−^ mice 1, although this study did not further categorize the cells based on CD103 expression. In contrast, in WT:Flt3^−/−^ mixed BM chimeras, the numbers of CD103^−^CD11b^+^ LP cDCs that derived from Flt3^−/−^ BM were only partially reduced compared with WT BM-derived CD103^−^CD11b^+^ LP cDCs 4.

## Antigen uptake and migration to MLNs by SI LP cDCs

### Sampling of luminal Ag by migratory LP cDC subsets

In their role as professional antigen presenting cells, intestinal DCs survey the content of the intestine and transport Ags to the MLNs where they initiate adaptive immune responses. Exactly how intestinal cDCs gain access to luminal Ag and whether intestinal cDC subsets sample soluble and particulate luminal Ags by similar routes is currently unclear. Initial studies suggested that CD11c^+^ cells could extend transepithelial dendrites (TEDs) through tight junctions in the epithelium to capture luminal bacteria 37. TED formation by CX3CR1^+^ cells was observed ex vivo in the terminal ileum of CX3CR1^+/gfp^ mice and this appeared to be CX3CR1-dependent 38. The functional relevance of these findings remains unclear, as although CX3CR1-dependent TEDs appeared to be required for uptake of invasion-deficient *Salmonella* 38, invasive *Salmonella* usually gain entry to the intestine primarily via microfold cells in PPs and ILFs 39–41 and CX3CR1-dependent TEDs were not required for the uptake of invasive *Salmonella* nor noninvasive *Aspergillus* spores 42. Additionally most CX3CR1^+^ cells in the intestine are tissue resident MΦs, rather than cDCs 1,2,43.

Two recent multiphoton microscopy studies used refined methods for identifying hematopoietic Ag sampling cells in the intestine 44,45. Both studies performed direct in vivo imaging of the intestine to help limit the potential artifacts that had been a concern in previous ex vivo approaches and CD11c^+/yfp^ x CX3CR1^+/gfp^ mice to allow distinction between CX3CR1^+^CD11c^+^ MΦs and CX3CR1^−^CD11c^+^ cDCs. McDole et al. 44 failed to detect TED extension by LP cDCs, but instead identified goblet cell-associated Ag passages that appeared to pass soluble dextran and proteins from the lumen to underlying GFP^−^YFP^hi^ cells. This route is unlikely to be involved in uptake of particulate Ag, such as luminal bacteria, as beads >0.02 μm diameter could not transit through GAPs. Similarly, Farache et al. 45 failed to observe LP cDCs extending TEDs but, in contrast to McDole et al., they noted a small population of CD103^+^ DCs within the epithelium itself, whose numbers increased in a CD103-and CCR6-independent manner following luminal administration of *Salmonella*. These intraepithelial CD103^+^ cDCs could be visualized extending intraepithelial dendrites into the lumen sampling *Salmonella* 45. These findings are in agreement with previous reports suggesting that low numbers of CD103^+^ cDCs are present in the epithelium at steady state, but increase during inflammation 11,46,47. Consistent with earlier work suggesting that orally administered protein Ag is taken up preferentially by CX3CR1^high^ MΦs 43, Farache et al. 45 found that intraepithelial CD103^+^ DCs were poor at capturing soluble Ag, compared with CX3CR1^high^ MΦs, which formed occasional TEDs. While the reason for the discrepant findings between the two studies regarding uptake of soluble Ag is not immediately apparent, Farache et al. 45 suggested it may reflect the ways in which the groups tuned their imaging systems to quantify YFP and GFP co-expression. McDole et al. 44 identified LP cDCs as GFP^−^YFP^high^ cells whereas Farache et al. 45 identified them as GFP^−^YFP^int^ cells and provided data to suggest that YFP^high^ cells were in fact CX3CR1^high^ MΦs. Clearly, further studies are required to assess the mechanisms by which intestinal cDCs acquire soluble and particulate derived luminal Ag, and whether the different LP cDC subsets perform overlapping or distinct roles in these processes.

### Migration of SI cDCs to draining MLNs

After acquiring Ag, intestinal cDCs migrate to draining MLNs in a CCR7-dependent manner 48–51. In earlier studies we provided strong, although indirect evidence that MLN CD103^+^ cDCs derive from the intestinal LP 49,52. By ex vivo confocal imaging of intestinal lymphatics and sampling of intestinal draining lymph, we 43 subsequently identified CD103^+^ DCs as the major migratory cDC population in intestinal lymph. More recently, by cannulating thoracic duct lymph from mesenteric lymphadenectomized mice, Cerovic et al. 5 confirmed that 75–85% of CD11c^+^MHCII^+^ cells in steady-state intestinal lymph are CD103^+^ cDCs. CD103^+^CD11b^+^ and CD103^+^CD11b^−^ cDCs were present in intestinal steady-state lymph at a ratio of ∼1.5–1, approximating that found in the MHCII^high^ migratory cDC population in MLNs. The remaining CD11c^+^MHCII^+^ cells were CCR7-expressing CD103^−^ cDCs, the majority of which were CD11b^+^ 5. Together, these data indicate that all subsets of bona fide LP cDCs migrate via afferent lymph to the MLN in the steady state.

Consistent with their lack of CCR7 expression and their absence from MLNs, CX3CR1^high^ MΦs were not found in intestinal draining afferent lymph in the studies by Schulz et al. 5,43 and Cerovic et al. 5,43. Contrary to these findings, Diehl et al. 53 recently reported CD103^−^CX3CR1^high^ cells in intestinal lymph in the steady state. While the reason for these discrepant findings is currently unclear, it is unlikely to be due to differences in the microbiota, as similar numbers of putative CX3CR1^high^ cells were observed in steady-state lymph following treatment of mice with broad spectrum antibiotics. One possible explanation is that the CX3CR1^high^ cells detected by Diehl et al. 53 are in fact CX3CR1^int^ cells and represent the CD103^−^ CD11b^+^ cDC subset of bona fide cDCs identified by Cerovic et al. 5.

## Role of intestinal cDC subsets in the initiation of adaptive immune responses

### LP cDCs in the priming of naïve CD4^+^ and CD8^+^ T cells

Migratory intestinal LP cDCs carry soluble and particulate Ags to the MLNs 11,54,55, where they are believed to play a critical role in the initiation of adaptive immune responses. Consistent with this idea, CD103^+^ cDCs isolated from the MLNs shortly after oral administration of soluble Ag induce Ag-specific proliferation of CD4^+^ and CD8^+^ T cells ex vivo 52,56 and CCR7-deficient mice fail to mount T-cell responses in MLNs after oral Ag administration 49,57. Recent studies analyzing FACS-purified cDC subsets from the SI, MLNs, and intestinal lymph suggest that all migratory cDC subsets can induce proliferation and functional polarization of naïve CD4^+^ T cells in vitro 5,13,24. In addition, consistent with their developmental relationship with lymphoid tissue CD8α^+^ DCs and CD103^+^CD8α^+^ DCs in other peripheral tissues 58–60, CD103^+^CD11b^−^ LP DCs appear superior to CD103^+^CD11b^+^ DCs at cross-presenting Ag to CD8^+^ T cells in vitro 5. Despite these findings, the role that particular intestinal migratory cDC subsets play in priming naïve CD4^+^ and CD8^+^ T cells in MLN in vivo is currently unclear, but could potentially be explored utilizing mice whose DCs are deficient in the distinct transcription factors described above.

### LP cDCs in the generation of gut-homing T cells

One of the characteristic features of the intestinal immune system is its anatomical compartmentalization, in which T and B cells primed in the GALT and MLNs are imprinted with the specific ability to return to the mucosa. In the case of the SI, this is dependent on the induction of the gut-homing molecules CCR9 and α4β7 on primed T cells, which are receptors for the chemokine CCL25 and the vascular adhesion molecule mucosal vascular addressin cell adhesion molecule 1 (MADCAM-1), respectively (for reviews see 61–63). Early studies by us and others demonstrated that small intestinal and MLN CD103^+^ cDCs display an enhanced ability to induce CCR9 and α4β7 on responding T cells in vitro, compared with DCs isolated from other tissues 49,52,64. Subsequently, MLN CD103^+^ cDCs were shown to have an enhanced ability to generate the vitamin A metabolite, retinoic acid (RA) 52,65,66, which is essential for gut-tropic T-cell generation in vitro and in vivo 65,67. Cerovic et al. 5 recently observed that all migratory cDC subsets including the small fraction of CD103^−^ cDCs in intestinal lymph metabolize vitamin A and induce gut-homing receptors on responding T cells in vitro, a finding that is consistent with the idea that migratory cDCs are imprinted with the ability to metabolize Vitamin A from signals they receive in the SI mucosa (for recent review see 68). In contrast to these in vitro studies, the role of SI DC-derived RA in the generation of gut-tropic T cells in vivo remains unclear. Thus, intestinal LP and MLN T cells from mice lacking Batf3-dependent CD103^+^CD11b^−^ MLN DCs have normal expression of CCR9 and α4β7 16, and we have recently shown that mice selectively lacking CD103^+^CD11b^+^ MLN DCs maintain their ability to induce gut-homing receptors on CD4^+^ T cells in vivo 24. These latter studies suggest that RA derived from either subset is sufficient to drive gut-tropic T-cell generation in vivo, or that there are other non-DC derived sources of RA, such as from MLN stromal cells 69,70, that fulfill this function.

### LP cDCs in Th cell differentiation

#### Inducible FoxP3^+^ Treg-cell generation

The MLN is a site that favors the differentiation of inducible FoxP3^+^ regulatory T (iTreg) cells 56,71. CCR7, FoxP3^+^ Treg cells, and MLNs are all required for the induction of tolerance to soluble luminal Ag 57,72, indicating a key role for migratory LP-derived cDCs in iTreg-cell generation. Consistent with this idea, small intestinal and MLN CD103^+^ cDCs display an enhanced capacity to generate iTreg cells from naïve precursors in vitro compared with other cDC subsets, in part through their ability to generate RA (56,71 and reviewed in 73). Mechanistically, RA alone fails to induce iTreg-cell differentiation but acts in synergy with TGF-β to enhance iTreg-cell generation in vitro. Indeed inhibition of RA receptor signaling prevents the enhanced capacity of CD103^+^ cDCs to drive Treg-cell differentiation in the presence of TGF-β 56,71,74. These original studies however did not distinguish between CD103^+^CD11b^+^ and CD103^+^CD11b^−^ DCs. Notably, DC-specific deletion of αvβ8, an integrin that activates latent TGF-β, leads to a reduction in FoxP3^+^ Treg-cell numbers and systemic autoimmunity 75 and CD103^+^CD11b^−^ cDCs appear to be the only intestinal cDCs that express αvβ8 (Scott and Persson, unpublished observations). Despite these findings, Batf3^−/−^ mice, which lack CD103^+^CD11b^−^ cDCs, have normal numbers of intestinal FoxP3^+^ Treg cells 16. We and others have also demonstrated recently that mice with a severe and selective reduction in migratory MLN CD103^+^CD11b^+^ cDCs have normal numbers of FoxP3^+^ Treg cells in the SI and colon 24,25, with equivalent ratios of Helios^+^ and neuropilin^+^ Treg cells when compared with WT mice 24. Interpretation of these results is complicated by a lack of clear markers to distinguish thymically derived natural Treg cells from iTreg cells in the intestinal mucosa. In fact, recent findings suggest that the majority of Treg cells in the intestine represent natural Treg cells 76,77 and thus the contribution of distinct intestinal migratory cDC subsets in driving iTreg-cell differentiation in MLN in vivo remains unclear.

#### Mucosal Th1/Th17 development

The steady-state intestinal LP contains a large pool of effector/memory CD4^+^ T cells with specificities against enteric microbial Ags and other luminal content 78; these CD4^+^ T cells include Th1 cells and the largest population of Th17 cells in the body. While it was thought initially that intestinally derived CD103^+^ cDCs preferentially induced Treg-cell responses 79,80, mounting evidence suggests that these DCs also play important roles in driving effector Th-cell differentiation in intestinal draining LNs. Moreover it appears that the individual subsets of intestinal cDCs may play distinct roles in promoting different arms of the mucosal adaptive immune response. In vitro studies indicate that both CD103^+^CD11b^+^ and CD103^+^CD11b^−^ cDCs can drive Th1 polarization 20, while CD103^+^CD11b^−^ SI DCs are effective at driving IFN-γ production by CD8^+^ T cells (5, our unpublished observations). Conversely, CD103^+^CD11b^+^ cDCs appear more efficient at inducing Th17 polarization than CD103^+^CD11b^−^ cDCs 13,20,24,81. More recently, CD103^−^ cDCs from intestinal steady-state lymph were suggested to be superior to both CD103^+^ subsets at inducing both Th1 and Th17 cells in vitro 5. We, and others, recently provided evidence that intestinal cDC subsets also play differential roles in the initiation of adaptive immune responses in vivo. Specifically, mice with a selective reduction in SI CD103^+^CD11b^+^ cDC numbers and an almost complete loss of these cells in intestinal draining MLNs, have reduced levels of endogenous Th17 cells in the SI, MLN, and colon 4,24, despite normal total CD4^+^ T-cell and Th1-cell numbers 24. Moreover we found that adoptively transferred CD4^+^ T cells failed to differentiate into Th17 cells in the MLNs of these mice following immunization with Ag and LPS and αCD40, while Th1-cell differentiation was unaffected. Mechanistically, it appeared that the selective absence of migratory CD103^+^CD11b^+^ cDCs in the MLNs resulted in reduced levels of IL-6, and that this was critical for driving Th17 differentiation in this location 24.

The in vivo signals that imprint DCs with the ability to generate effector T cells in the healthy intestine are unclear, although a role for pattern recognition receptors including toll-like receptors (TLRs) and MyD88 signaling has been suggested 82,83. Indeed ligands for several TLRs including TLR2, TLR5, and TLR9 induce IL-6 production by CD103^+^CD11b^+^ DCs in vitro and promote their ability to generate Th17-cell differentiation 5,20,24. In this regard, it is interesting to note that CD103^+^CD11b^+^ cDCs and CD103^+^CD11b^−^ cDCs express a partially overlapping and partially distinct array of pattern recognition receptors 20,81,84,85, suggesting that they may respond differentially to microbial signals in the intestinal lumen.

## Summary and concluding remarks

Intestinal DCs are a crucial link between the innate and adaptive immune responses and thus provide an attractive target for therapeutic manipulation of the intestinal immune response. Although similar levels of CD11c and MHCII expression by intestinal DCs and MΦs have impeded reliable characterization of DCs in the past, recent advances in phenotyping now allow these cells to be more precisely discriminated. As a result, it is now believed that there are at least three populations of bona fide DCs in the murine intestinal LP: two CD103-expressing cDC subsets, CD103^+^CD11b^+^ and CD103^+^CD11b^−^ cDCs, which derive from committed cDC precursors; and a more recently described CD103^−^CD11b^+^ population whose functions and origins remain to be characterized fully. The recent development of specific knock out mouse models with selective reduction in individual intestinal cDC populations has also allowed researchers to start addressing the functions of individual intestinal cDC subsets in the regulation of adaptive immune responses in vivo. Initial studies with these mice have demonstrated that the previous assertions that CD103^+^ cDCs were inherently tolerogenic are incorrect, and that, for example intestinal CD103^+^CD11b^+^ cDCs play an important role in driving Th17 differentiation in the MLNs 24. It remains unclear if each cDC subset is hardwired to generate specific aspects of the T-cell pool in vivo, or if plasticity of the subsets can allow each to drive different forms of T-cell responses as required. If so, it seems likely that this plasticity will be determined by signals from the local environment. As well as identifying the factors involved, it will be important to determine at what stage they act in cDC development. For instance, do local signals act on newly arrived precursors and predetermine the functions of their progeny, or do mature cDCs adjust when they encounter different contexts in the tissues? Finally it is important to note that progress has also been made in identifying cDC subsets in the human intestinal LP. The majority of cDCs in the healthy SI also express CD103 and can be similarly divided into two subsets 24, a major population of SIRPα^+^ IRF4-expressing cDCs that we speculate are equivalent to murine intestinal CD103^+^CD11b^+^ DCs, and a minor population of CD141^+^ cDCs that are likely equivalents of CD103^+^CD11b^−^ DCs 86–88. It remains to be seen if an equivalent to murine CD103^−^ cDCs exists in the human intestine. Determining whether these human intestinal cDC subsets function in a similar way to their murine counterparts will be important if these cells are to be exploited for the treatment and/or prevention of human inflammatory bowel diseases and in the development of mucosal vaccines.

## Abbreviations

Batf3: basic leucine zipper transcription factor ATF-like 3
cDC: classical dendritic cell
Flt3L: FMS-like tyrosine kinase 3 ligand
Id: inhibitor of DNA-binding
ILF: isolated lymphoid follicle
IRF: IFN-regulatory factor
iTreg: inducible FoxP3^+^ regulatory T
LP: lamina propria
MP: mononuclear phagocyte
PP: Peyer's patch
RA: retinoic acid
SI: small intestine
SIRP: signal regulatory protein
TED: transepithelial dendrite
